# “Placement Budgets” for Supported Employment—Impact on Quality of Life in a Multicenter Randomized Controlled Trial

**DOI:** 10.3389/fpsyt.2018.00462

**Published:** 2018-09-26

**Authors:** Wulf Rössler, Wolfram Kawohl, Carlos Nordt, Helene Haker, Nicolas Rüsch, Michael P. Hengartner

**Affiliations:** ^1^Department of Psychiatry, Psychotherapy and Psychosomatics, University of Zurich, Zurich, Switzerland; ^2^Department of Psychiatry and Psychotherapy, Charité Universitätsmedizin Berlin, Berlin, Germany; ^3^Laboratory of Neuroscience (LIM 27), Institute of Psychiatry, University of São Paulo, São Paulo, Brazil; ^4^Department for Psychiatry and Psychotherapy, Psychiatric Services Aargau, Brugg, Switzerland; ^5^Translational Neuromodeling Unit, Institute for Biomedical Engineering, University of Zurich and ETH Zurich, Zurich, Switzerland; ^6^Department of Psychiatry II, University of Ulm and BKH Günzburg, Günzburg, Germany; ^7^Department of Applied Psychology, Zurich University of Applied Sciences, Zurich, Switzerland

**Keywords:** psychiatric rehabilitation, quality of life, supported employment, randomized trial, severe mental disorder

## Abstract

**Background:** Employment is an important aspect of psychiatric rehabilitation. The objective of this analysis was to explore how quality of life (QoL) may affect the outcome of supported employment and vice versa.

**Methods:** A total of 116 participants with severe mental disorders were randomly assigned to either 25, 40, or 55 h placement budgets, which comprises job coaches' time resources to support a client in finding a job. The intervention followed the individual placement and support model and lasted up to 36 months. Primary outcome was employment in the first labor market for at least 3 months. QoL was assessed 7 times over the entire 36-months observation period using the WHO QoL Bref, which comprises the dimensions physical health, psychological, social relationships, and environment.

**Results:** The three placement budgets did not differentially relate to QoL, but QoL environment showed a significant increase over time across all three groups. Baseline QoL environment weakly predicted subsequent obtainment of employment (*F* = 4.08, *df* = 1, *p* = 0.046, Cohen's *d* = 0.39). Controlling for baseline QoL, those participants who obtained a job, as compared to those who did not, showed persistent increases in QoL physical health (*b* = 0.39, *p* = 0.002, Cohen's *d* = 0.50) and QoL psychological (*b* = 0.40, *p* < 0.001, Cohen's *d* = 0.47).

**Conclusion:** Obtaining employment in the first labor market improves patients' QoL. Supported employment is a valuable intervention that may benefit patients with severe mental disorder.

## Introduction

Psychiatric rehabilitation aims to help persons with serious and persistent mental disorders to live a normal life in the community with the least amount of professional support (1). As the majority of mental disorders begins during adolescence, early or middle adulthood (2), a work career is regularly a central part of the life plans of those people affected. But unfortunately unemployment is widespread among persons with severe mental illness. In Western industrialized countries, unemployment rates between 60 and 90% were published, e.g., for treatment-seeking people with psychotic disorders (3, 4). In people with major depression unemployment rates appear to be lower (5, 6), but presenteeism (working while being ill) and absenteeism (missing at work due to illness) are common (7, 8). Worldwide the unemployment gap between persons with mental disorders and unaffected persons between the mid-1990s and the mid- to late 2000s has even increased (9).

In Switzerland, around 30% of the working-age population with a moderate or severe mental disorder was unemployed in 2007, which is the lowest unemployment rate among all OECD countries (10). Treatment-seeking people with chronic mental disorders of course have much higher unemployment rates and are more likely to be out of the labor force. Consequently, sickness and disability benefit spending is high in Switzerland, and by 2012, mental disorders accounted for about 37% of all disability benefits (10).

Therefore, vocational rehabilitation has been a core element of psychiatric rehabilitation since its beginning (1). Vocational rehabilitation is based on the assumption that work does not only improve activity, but also structures the day, constitutes social contacts, provides social support as well as it increases self-esteem, and finally is a step away from dependency. All these elements are incorporated in the concept of quality of life (QoL) in persons with mental disorders (11, 12). Currently, the most promising vocational rehabilitation model is supported employment (SE) (1, 13). The “Individual Placement and Support model” (IPS), as conceptualized by Becker and Drake (14), is a cornerstone of SE and helps persons with serious and persistent mental disorders to find competitive employment according to their choices as soon as possible and receive all support needed to maintain their position. Participation in SE programs is followed by an increase in the ability to find and keep employment (15, 16). Links were also found between job tenure and non-vocational outcomes, such as improved self-esteem, social integration, and relationships. With respect to competitive employment, the benefit of IPS compared to traditional vocational rehabilitation was confirmed in two recent meta-analyses (13, 17). Here we want to expand this literature by focusing on associations between competitive employment and QoL.

Although findings regarding SE are encouraging, some critical issues remain to be answered. In the original IPS model, support provided to find and keep employment is continued indefinitely. However, one of the major barriers for introducing SE in standard care in European countries is the unlimited provision of SE, which does not comply with health and social service legislation in many European countries (18). Since the main principle of IPS is “place first, then train,” one could argue that constraints on the time budget for selection of a job could impose stress on the participant. This, in turn, could have a negative impact on QoL, which is why it is important to explore how different placement budgets relate to QoL. Another issue that we wanted to address is whether obtaining competitive employment within SE translates into improved QoL. In our multicenter RCT we therefore tested the impact of different “placement budgets,” i.e., a pre-defined time budget with a maximum number of hours of help provided for job search (25, 40, or 55 h) on the QoL of the participants in this trial.

In the present work, we addressed the following three research questions: (i), do different placement budgets have an effect on QoL, (ii), do baseline QoL scores have an effect on the probability of obtaining a competitive employment, and (iii), has obtaining a competitive employment an effect on subsequent QoL scores?

## Methods

### Participants and procedure

**Figure 1:**
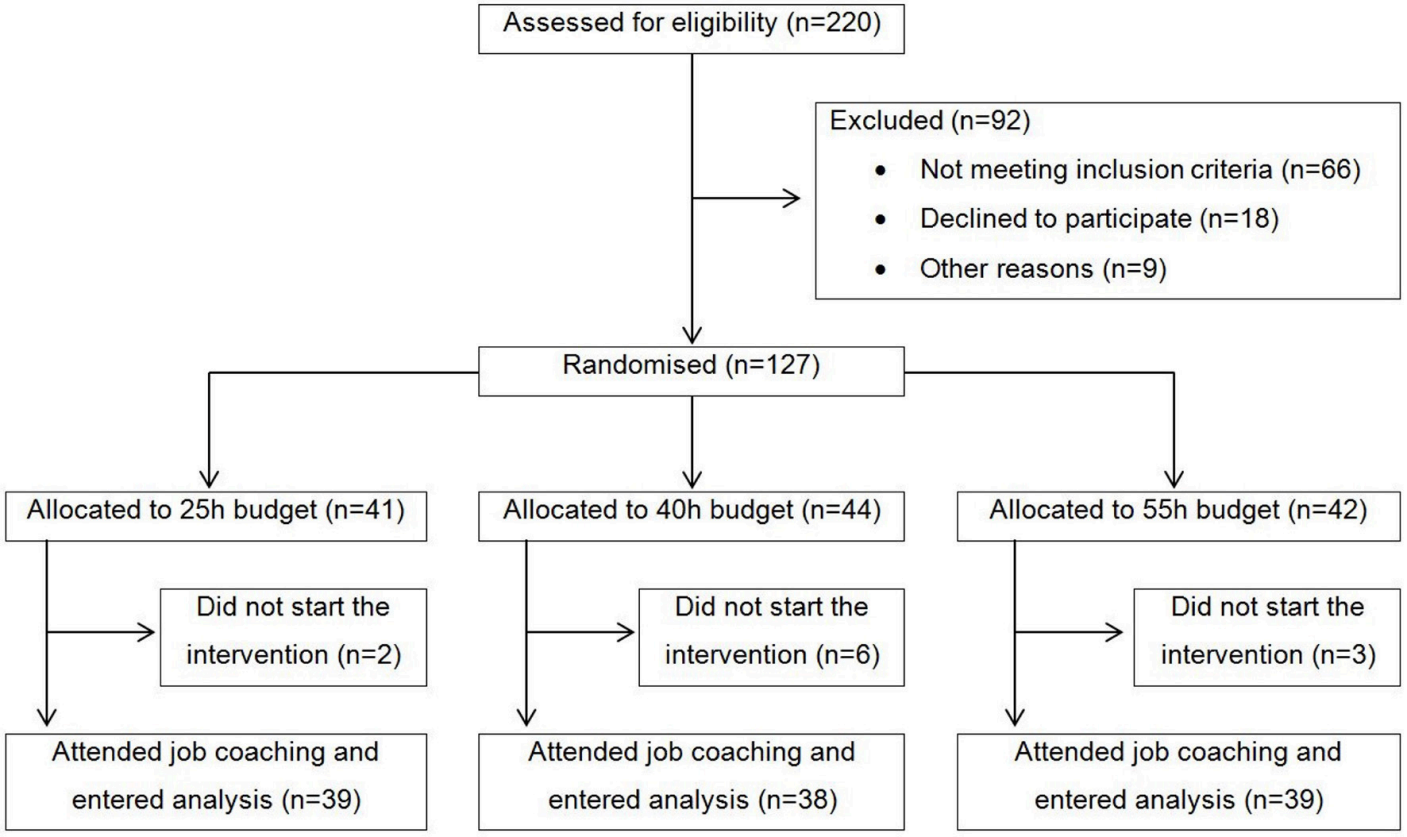
Participant flow.

Participants were recruited from six outpatient clinics in the canton of Zurich, Switzerland, between June 2010 and May 2011. Participants were enrolled by six different job coaches, which subsequently also provided support for the search of a job in the first labor market. Inclusion criteria were current treatment in one of the six participating psychiatric outpatient clinics, at least 1 year of unemployment, no participation in a vocational integration programme during the last 3 months, being of working age (i.e., 18–60 years), a desire to obtain competitive employment in first job market, being willing, and capable of giving informed consent, and resident of the canton of Zurich. Exclusion criteria were severe organic illness und insufficient knowledge of German language. All participants provided written informed consent. The CONSORT flow chart is shown in Figure 1. Altogether *n* = 116 participants started the intervention and were included in our intent-to-treat analysis.

The Psychiatric University Hospital has its own organizational unit for SE. This unit has developed a formal application process for potential coaches, which includes the formal job requirements as well as the personal skills needed for job coaches. Job coaches were trained in the IPS model and had weekly meetings with supervision at the above mentioned Supported Employment Unit of the Psychiatric University Hospital of Zurich. The specific intervention chosen for this trial is the Individual Placement and Support (IPS) model (14, 19). The job coaches supported the clients for up to 2 years, or until the placement budget had run out for those who failed to find competitive employment.

For the present trial a web based software programme was tailored with a schedule tool for job coaches that automatically computed their remaining placement budgets. In accordance with the IPS model, the job coaches assisted the participants in the following two tasks. First, placement (engagement, assessment, and to find a job that matches a client's skills and interests); and second, support (help to maintain competitive employment). The time-restriction applied to assistance for job search only. That is, independent of the assignment to different placement budgets, all participants received unlimited support for job maintenance once they successfully started competitive employment. Implementation fidelity was assessed every 3 months with the supported employment fidelity scale (14).

Altogether 7 assessments were conducted, one baseline assessment (t0) and six follow-ups (t1–t6), which were conducted every 6 months for a total observation period of 36 months. Retention was good, with *n* = 86 (74%) participants participating in the 24 months follow-up and *n* = 77 (66%) participating in the 36 months follow-up.

### Randomization

Block randomization with a block size of six was chosen, so that for each job coach, all three budget groups were similarly sized. Participants were assigned to either 25, 40, or 55 h placement budgets based on a 1:1:1 allocation ratio. We used PROC FACTEX from SAS to generate a random order for the placement budgets, which was applied in each participating outpatient clinic. There was no allocation concealment mechanism in place. Allocation to placement budget could not be blinded to both participants and job coaches, because job coaches and participants had to discuss how best to invest the allocated budget.

### Ethics

The trial was pre-registered in ISRCTN registry (trial number: ISRCTN89670872) and the study protocol was published freely available online (20). The study protocol was approved by the Zurich Cantonal Ethics Committee (CEC), reference number E-51/2009.

### Instruments and measures

Irrespective of whether participants were still supported by their job coach, trained research assistants carried out assessments every 6 months over a total period of 3 years via computer-assisted face-to-face interviews. Employment status, duration of employment and job description over the last 6 months were assessed via participants' self-report.

QoL was assessed with the German translation of the World Health Organization Quality of Life Bref [WHO QoL Bref; (21)]. This self-report questionnaire captures QoL based on four dimensions, specifically (1) physical health (e.g., dependency on medical aids, energy and fatigue, mobility, work capacity), (2) psychological (e.g., negative and positive feelings, self-esteem, memory, and concentration), (3) social relationships (e.g., personal relationships, social support, sexual activity), and (4) environment (e.g., financial resources, home environment, physical environment, safety, and security). The WHO QoL has been shown to be a valid and reliable measure of QoL (21, 22).

Socio-demographic and clinical characteristics at baseline were derived from the German version of the Client Socio-demographic and Service Receipt Inventory [CSSRI; (23)] and from the Central Psychiatric Register of the Canton of Zurich (PSYREC), which includes the following information from clinical records: diagnosis according to ICD-10, Global Assessment of Functioning (GAF) score (24), and Clinical Global Impression (CGI) scale (25).

### Statistical analysis

For the longitudinal analysis of repeated measures of QoL we fitted a series of Generalized Estimating Equations (GEE) (26), where QoL dimensions were included separately as the dependent variable. GEE models were introduced to fit regression analyses that account for within-subject correlation, which is an inherent part of longitudinal studies that rely on repeated outcome measures. The GEE approach uses weighted combinations between a predictor variable and repeated outcomes that account for varying observations, e.g., QoL being high or low, within a person across time. GEE use all available data and estimate missing values under the assumption of Missing Completely at Random (MCAR) (27). Prerequisite to the application of GEE was therefore a thorough missing value analysis, which confirmed that QoL psychological, social relationships, and environment all met the criteria of MCAR according to Little's MCAR test (all *p* > 0.3), whereas QoL physical health did not (X^2^ = 100.8, *df* = 77, *p* = 0.036). However, as there were only 23 (19.8%) missing values at 18 months follow-up, 30 (25.9%) at 24 months follow-up and 39 (33.9%) at 36 months follow-up out of a total sample size of *n* = 116, we determined that any bias due to violation of the MCAR assumption would be minor. Based on the probability density function of the QoL dimensions we applied normal distribution with identity link-function. The within-subject covariance was specified with the “unstructured” correlation type to avoid having any constraints on the covariance structure and a robust sandwich estimator was used to reduce the effects of outliers and influential observations. All analyses were conducted with SPSS version 24 for Windows.

## Results

Socio-demographic and clinical characteristics of study participants according to randomly allocated placement budgets are indicated in Table 1. Only F6 diagnosis differed significantly between groups, with a low frequency in the 25 h group, intermediate frequency in the 40 h group, and a relatively high frequency in the 55 h group.

**Table 1:** Baseline characteristics across study groups.

	**25 h budget *N* = 39**	**40 h budget *N* = 38**	**55 h budget *N* = 39**	**Statistical test of group differences**
**Continuous variables**	**Mean (*****SD*****)**	**Mean (*****SD*****)**	**Mean (*****SD*****)**	
Age (in years)	40.8 (10.4)	41.7 (10.4)	41.3 (10.7)	*F* = 0.06, *df* = 2; *p* = 0.943
Education (in years)	11.8 (3.5)	11.5 (3.2)	10.9 (2.3)	*F* = 0.91, *df* = 2; *p* = 0.404
GAF Score	57.3 (10.5)	56.7 (11.7)	54.2 (13.79	*F* = 0.73, *df* = 2; *p* = 0.486
CGI Score	4.8 (1.0)	4.7 (1.4)	5.1 (0.8)	*F* = 1.76, *df* = 2; *p* = 0.176
**Categorical variables**	**N (%)**	**N (%)**	**N (%)**	
Female gender	23 (59.0)	19 (50.0)	17 (43.6)	X^2^ = 1.86, *df* = 2; *p* = 0.394
F1 diagnosis	4 (10.3)	3 (7.9)	5 (12.8)	X^2^ = 0.50, *df* = 2; *p* = 0.777
F2 diagnosis	3 (7.7)	4 (10.5)	4 (10.3)	X^2^ = 0.22, *df* = 2; *p* = 0.895
F3 diagnosis	22 (56.4)	15 (39.5)	13 (33.3)	X^2^ = 4.54, *df* = 2; *p* = 0.103
F4 diagnosis	8 (20.5)	9 (23.7)	4 (10.3)	X^2^ = 2.57, *df* = 2; *p* = 0.277
F6 diagnosis	1 (2.6)	6 (15.8)	9 (23.1)	X^2^ = 7.09, *df* = 2; *p* = 0.029

*GAF, Global Assessment of Functioning scale; CGI, Clinical Global Impressions scale; F1, Mental and behavioral disorders due to psychoactive substance use; F2, Schizophrenia, schizotypal, delusional, and other non-affective psychotic disorders; F3, Affective disorders; F4, Anxiety, dissociative, stress-related, somatoform and other non-psychotic mental disorders; F6, Disorders of adult personality and behavior*.

The association between QoL and placement budgets are shown in Table 2. According to a series of GEE analyses conducted for each QoL dimension separately, placement budget was associated with no QoL dimension (all *p* > 0.3). However, there was a significant effect for time on QoL environment (Wald X^2^ = 14.922, *df* = 5, *p* = 0.011), indicating that QoL environment increased steadily from t1 to t6 in the full sample.

**Table 2:** Randomly allocated placement budgets in association with repeated measures of QoL.

**Time point**	**QoL dimension**	**25 h budget**	**40 h budget**	**55 h budget**	**Total**
Baseline (t0)	Physical health Psychological Social relationships Environment	3.39 (0.73) 2.98 (0.78) 3.16 (0.85) 3.61 (0.59)	3.38 (0.74) 3.03 (0.74) 3.32 (0.82) 3.60 (0.46)	3.34 (0.73) 3.00 (0.82) 3.17 (0.87) 3.56 (0.51)	3.37 (0.73) 3.00 (0.77) 3.22 (0.84) 3.59 (0.52)
6 months (t1)	Physical health Psychological Social relationships Environment	3.53 (0.79) 3.28 (0.83) 3.49 (0.59) 3.64 (0.54)	3.38 (0.80) 3.23 (0.77) 3.31 (0.85) 3.74 (0.53)	3.50 (0.76) 2.95 (0.87) 3.29 (0.88) 3.59 (0.47)	3.47 (0.78) 3.15 (0.83) 3.36 (0.79) 3.66 (0.51)
12 months (t2)	Physical health Psychological Social relationships Environment	3.58 (0.91) 3.51 (0.87) 3.47 (0.86) 3.75 (0.62)	3.50 (0.70) 3.27 (0.65) 3.51 (0.78) 3.74 (0.48)	3.51 (0.67) 3.13 (0.73) 3.42 (0.74) 3.67 (0.53)	3.53 (0.76) 3.31 (0.77) 3.47 (0.79) 3.72 (0.54)
18 months (t3)	Physical health Psychological Social relationships Environment	3.65 (0.89) 3.45 (0.96) 3.52 (0.90) 3.80 (0.56)	3.73 (0.70) 3.39 (0.67) 3.38 (0.78) 3.74 (0.46)	3.41 (0.84) 3.21 (0.85) 3.37 (0.93) 3.70 (0.50)	3.59 (0.81) 3.35 (0.83) 3.42 (0.87) 3.75 (0.50)
24 months (t4)	Physical health Psychological Social relationships Environment	3.59 (0.97) 3.53 (0.91) 3.52 (0.95) 3.87 (0.62)	3.65 (0.52) 3.47 (0.63) 3.56 (0.75) 3.77 (0.40)	3.56 (0.68) 3.27 (0.84) 3.46 (0.74) 3.71 (0.54)	3.60 (0.74) 3.42 (0.80) 3.51 (0.81) 3.78 (0.53)
30 months (t5)	Physical health Psychological Social relationships Environment	3.74 (0.81) 3.55 (1.00) 3.55 (0.94) 3.88 (0.70)	3.66 (0.70) 3.44 (0.77) 3.50 (0.78) 3.91 (0.57)	3.41 (0.84) 3.23 (0.97) 3.46 (0.80) 3.81 (0.49)	3.61 (0.78) 3.41 (0.92) 3.50 (0.83) 3.87 (0.59)
36 months (t6)	Physical health Psychological Social relationships Environment	3.72 (0.85) 3.71 (0.75) 3.43 (0.71) 3.86 (0.57)	3.71 (0.75) 3.38 (0.64) 3.34 (0.77) 3.85 (0.45)	3.43 (0.71) 3.32 (1.07) 3.44 (0.89) 3.88 (0.52)	3.59 (0.81) 3.47 (0.84) 3.40 (0.78) 3.86 (0.51)

*Results are indicated with mean scores and standard deviations (in brackets)*.

A total of *n* = 46 (39.7%) participants obtained a competitive employment in first job market. We therefore tested whether baseline QoL (t0) would relate to subsequent obtainment of a competitive employment based on a series of one-factorial ANOVA. Only baseline QoL environment differed significantly (*F* = 4.081, *df* = 1, *p* = 0.046) between those who later obtained a job and those who did not (job obtained: *m* = 3.71, *SD* = 0.51; no job: m = 3.51, *SD* = 0.51, Cohen's *d* = 0.39). Since all jobs were obtained between months 1 and 23 (t1–t3), we used job obtainment (yes vs. no) as a predictor of QoL from month 24 to 36 (t4–t6), while controlling for temporal stability (assessment time t4–t6) and baseline QoL (t0). The results of these GEE analyses are reported in Table 3. Obtaining a competitive job between t1 and t3 prospectively predicted QoL physical health (*b* = 0.39, *p* = 0.002, Cohen's *d* = 0.50) and QoL psychological (*b* = 0.40, *p* < 0.001, Cohen's *d* = 0.47) from t4 to t6. These associations were independent of baseline QoL and temporally stable, indicating that job-related gains in QoL were preserved over time. Adding baseline CGI scores to the model did not alter the results.

**Table 3:** Prospective effect of obtaining a competitive job on subsequent QoL, controlling for temporal stability and baseline QoL.

**QoL dimension**	**Predictor**		**QoL t4 through t6**	
			**B (95% CI)**	***P***
Physical health	Competitive job	Yes No	0.39 (0.15; 0.63) Reference	0.002
	Time point	T6 T5 T4	−0.02 (-0.14; 0.10) 0.02 (-0.09; 0.13) Reference	0.746 0.727
	Physical health at baseline		0.48 (0.32; 0.64)	<0.001
Psychological	Competitive job	Yes No	0.40 (0.17; 0.62) Reference	<0.001
	Time point	T6 T5 T4	(−0.11; 0.12) (−0.11; 0.11) Reference	0.933 0.962
	Psychological at baseline		0.70 (0.57; 0.83)	<0.001
Social relationships	Competitive job	Yes No	0.23 (−0.02; 0.48) Reference	0.067
	Time point	T6 T5 T4	−0.10 (−0.27; 0.07) 0.05 (−0.09; 0.19) Reference	0.253 0.485
	Physical health at baseline		0.47 (0.29; 0.65)	<0.001
Environment	Competitive job	Yes No	0.15 (−0.01; 0.31) Reference	0.060
	Time point	T6 T5 T4	0.09 (−0.01; 0.20) 0.09 (−0.01; 0.18) Reference	0.089 0.064
	Physical health at baseline		0.42 (0.23; 0.61)	<0.001

## Discussion

QoL is an important outcome measure in the provision of mental health care. In this study, we examined how different placement budgets may affect QoL and whether obtaining a competitive employment had an effect on QoL. Our data revealed several interesting findings. First, changes in QoL over the total observation period of 36 months did not differ in relation to smaller or larger placement budgets, but overall it was found that QoL environment increased over time. Second, baseline scores in QoL environment weakly predicted subsequent obtainment of a competitive employment. Third, patients who obtained a competitive employment, compared to patients who did not, showed subsequent increases in QoL physical health and QoL psychological that were independent of baseline QoL scores and which persisted over time.

Thus, the fact that there is a placement budget randomly allocated to each participant does not result in an improvement or a deterioration of the participants' QoL. In fact, critics of such placement budgets would rather argue that a restricted provision of care time would place an undue pressure and stress on participants and rather deteriorate than improve their overall situation. But this is not the case even for the smallest placement budget. On the contrary, over six assessment points there is an improvement of the participants' QoL. It seems that entering such a SE program gives increasingly hope to the participants, as “hope” is a strong force in the rehabilitation process (1, 18).

QoL environment baseline values predicted weakly whether an individual obtains an employment. This does not really come as a surprise as individuals whose environmental resources are better, also have better chances on the labor market. Interestingly, the other QoL dimensions did not significantly predict successful employment. These findings suggest that with appropriate support and motivation most patients could overcome initial deficits in QoL dimensions such as physical health, psychological resources, and social relationships.

And finally, it is important to note that individuals obtaining an employment, experience a persistent improvement of their psychological and physical QoL compared to those who did not. This is a strong argument for the integration of persons with mental illness into society, i.e., not to separate and care for them in traditional specialized services as we do today in sheltered workshops. This is particularly relevant in the light of their young age and the chronic character of the disorders they suffer from. An alternative explanation would be that receiving support from a trained job coach improves QoL rather than the competitive employment. More likely, however, is an interaction between the effects of receiving support and having a competitive employment. In future research it would thus be worthwhile to focus on the different mechanisms that may account for an association between employment and QoL (e.g., autonomy, empowerment, self-esteem, interpersonal contact).

### Strengths and limitations

The present work offers several strengths, such as thorough randomization to different placement budgets, repeated assessment of QoL, a work outcome which exceeds the minimum time of employment compared to most other studies in the field, and a long-term follow-up with high retention rate. However, we also acknowledge the following two limitations. First, and most importantly, QoL relied on self-report and might be influenced by experienced acute distress and psychopathological symptoms. As a result, changes in QoL scores do not necessarily reflect true change in QoL. An alternative explanation would be that changes merely reflect an altered subjective perception of one's QoL. These findings thus require replication with objective indicators of QoL. Second, owing to small number of participants, the representativity of the sample is uncertain. This may also restrict the generalizability of our findings to some unknown degree.

## Conclusion

Successfully obtaining competitive employment through SE appears to significantly improve patient's QoL, even when placement budgets are restricted. SE therefore is a useful and cost-effective intervention (28) that may benefit people with severe mental disorders in the long run. More research is required to determine causal mechanisms that are involved.

## Author contributions

WR and MH took responsibility for the statistical analyses and drafted and revised the manuscript. CN and WK took responsibility for the study design, supervised the assessments, and substantially contributed to drafting and critical revision of the manuscript. HH and NR took responsibility for special assessments and substantially contributed to drafting and critical revision of the manuscript.

### Conflict of interest statement

The authors declare that the research was conducted in the absence of any commercial or financial relationships that could be construed as a potential conflict of interest. The reviewer JS and handling editor declared their shared affiliation at the time of the review.
